# Tollip or Not Tollip: What Are the Evolving Questions behind It?

**DOI:** 10.1371/journal.pone.0097219

**Published:** 2014-05-14

**Authors:** Denis Prudencio Luiz, Célio Dias Santos Júnior, Ana Maria Bonetti, Malcom Antônio Manfredi Brandeburgo

**Affiliations:** Genetics Laboratory, Genetics and Biochemistry Institute, Federal University of Uberlandia - UFU, Uberlândia, Minas Gerais, Brazil; Virginia Polytechnic Institute and State University, United States of America

## Abstract

Tollip plays an important role in the interleukin-1 receptor IL-1R and Toll pathways. As a modulator of the immune pathway, it indirectly controls the amount of antimicrobial peptides. This could indicate a vital step in maintaining animal immune systems and preventing infection. Evolutionary questions are crucial to understanding the conservation and functioning of the biochemical pathways like the Tollip-mediated one. Through an analysis of 36 sequences of the Tollip protein from different animal taxa, downloaded from Kyoto Encyclopedia of Genes and Genomes (KEGG) databank, we inferred diverse evolutionary parameters, such as molecular selection and structure conservation, by analyzing residue by residue, beyond the canonical parameters to this type of study, as maximum likelihood trees. We found that Tollip presented different trends in its evolving history. In primates, the protein is becoming more unstable, just the opposite is observed in the arthropod group. The most interesting finding was the concentration of positively selected residues at amino terminal ends. Some observed topological incongruences in maximum likelihood trees of complete and curated Tollip data sets could be explained through horizontal transfers, evidenced by recombination detection. These results suggest that there is more to be researched and understood about this protein.

## Introduction

There is a lot of biological information deposited in online databases, but little of the data is analyzed properly [1], [2]. These data are largely used in bioinformatics, covering various areas such as computer science, mathematics and biological engineering several. Thus it is possible to optimize these studies, in a simple way [3]. The bioinformatic data can be used in phylogenetic analysis, as it is used in most branches of biology, such as phylogenetic trees for paralog genes [4], population analysis [5], evolution, epidemiology [6], [7], and genomic and metagenomic sequence comparison [8]. Protein phylogeny is used to indicate synonymous and non-synonymous substitutions along with the branches in order to identify cases of rapid changes of amino acids [9]. The analysis of different trees allows for the observation of topological incongruences, differences in the formation of taxa, and the relationship between nodes and trees [10], [11]. The complete phylogenetic inference at species level is presented in the Tree of Life (ToL) Web Project. ToL is a collaborative project of hundreds of phylogenetic researchers correlating diverse sources of information, including morphological, physiological, and molecular information. (This project is a work in progress [12]).

The presence of pathogens in the environment can interfere in the survival and reproduction of individuals in a population, leading to new evolutionary trends [13], [14]. Multicellular organisms have a rapid immune response to pathogens entering, named innate immunity. This response is performed by specialized cells, which have specific receptors for pathogen-associated molecular patterns (PAMPs) [15], [16], the most noticeable are Toll-Like Receptors (TLRs) [17]. Tollip (Toll-interacting protein) participates in the signaling pathway of the TLR with an endogenous modulatory role. Tollip has a target N-terminal Myb1 (Tom1) binding domain (TBD), a conserved core domain 2 (C2) and a C-terminal portion of coupling ubiquitin to endoplasmic reticulum degradation (CUE). In resting cells, Tollip controls the activation pathway of Myeloid differentiation primary response gene (88) (MyD88)-dependent NF-kB in two different ways. First, Tollip associates with IL-1R, TLR4 after LPS activation, inhibiting the immune response mediated by TLR [18], [19]. This association requires TLR-TIR domain and intact C-terminal region of Tollip, CUE domain. Second, Tollip binds directly to interleukin-1 receptor-associated kinase-1 (IRAK-1) by inhibiting an autophosphorylation but without promoting its degradation. Overexpression of Tollip leads to inhibition of TLR2, TLR4, and IL-1R signaling, confirming a modulatory role of Tollip in immune responses [20]–[23].

The main goal of this paper is to show the topological incongruences between Tollip protein sequence phylogenetic trees using ToL data as reference. Other goals are to determine the diversity in the evolution of this protein in different taxa, the possible horizontal gene transfers, and the correlation of molecular features in the sequences within primates and arthropod groups.

## Material and Methods

Thirty-six sequences of Tollip protein were downloaded from KEGG (Table 1). The phylogenetic reference used was the Tree of Life Web Project, ToL (http://tolweb.org/tree/), which were used in topological comparisons with Tollip generated data.

**Table 1:** Tollip downloaded reference data from KEGG and principal protein features.

Specie	Code	Tollip protein N. of access	pI	MW (Da)	Instability Index	Aliphatic Index	GRAVY
*Anolis carolinensis*	acs	100562725	4.99	31238.86	54.21	79.6	−0.295
*Anopheles gambiae*	aga	AGAP003615	5.37	30424.6	42.29	85.57	−0.319
*Apis mellifera*	ame	552034	5.63	31855.55	55.02	79.36	−0.334
*Ailuropoda melanoleuca*	aml	100463737	4.75	35361.18	47.73	83.28	−0.212
*Acyrthosiphon pisum*	api	100167693	5.31	30628.78	37.88	83.79	−0.453
*Amphimedon queenslandica*	aqu	100616078	5.55	33666.77	47.75	66.31	−0.706
*Bos taurus*	bta	539480	5.3	30107.58	43.7	84.25	−0.191
*Canis familiaris*	cfa	483657	5.05	30068.36	44.38	81.79	−0.211
*Ciona intestinalis*	cin	100178902	4.96	33008.67	41.76	77.63	−0.438
*Danio rerio*	dre	336876	4.93	30435.85	45.65	80.54	−0.232
*Equus caballus*	ecb	100051383	6.02	45675.89	51.53	77.7	−0.408
*Felis catus*	fca	101095614	5.18	29976.31	42.47	82.52	−0.186
*Gallus gallus*	gga	423099	5.03	30539.94	48.15	78.61	−0.317
*Gorilla gorilla gorilla*	ggo	101153660	6.51	32640.55	51.84	82.73	−0.134
*Hydra magnipapillata*	hmg	100204873	4.91	24690.68	42.49	85.37	−0.226
*Homo sapiens*	hsa	54472	5.68	30281.81	54.72	82.19	−0.243
*Ixodes scapularis*	isc	ISCW002962	6.79	21534.59	24.65	92.49	−0.073
*Macaca mulatta*	mcc	702587	5.68	30311.83	54.72	81.82	−0.252
*Monodelphis domestica*	mdo	100011810	5.04	30174.46	48	84.31	−0.232
*Meleagris gallopavo*	mgp	100543926	4.89	31330.93	46.17	82.14	−0.279
*Mus musculus*	mmu	54473	5.04	30344.82	52.35	76.86	−0.29
*Nasonia vitripennis*	nvi	100115445	5.62	31502.04	45.79	81.29	−0.4
*Ornithorhynchus anatinus*	oaa	100078225	8.9	23160.41	47.24	72.92	−0.498
*Oryzias latipes*	ola	101163379	5.05	30281.61	46.64	81.92	−0.192
*Pediculus humanus corporis*	phu	PHUM008100	5.8	29271.21	42.43	82.81	−0.362
*Pongo abelii*	pon	100442943	5.68	30281.81	54.72	82.19	−0.243
*Pan paniscus*	pps	100994067	5.08	22812.3	62.26	79.41	−0.229
*Pan troglodytes*	ptr	450943	5.79	63790.54	53.35	71.86	−0.263
*Rattus norvegicus*	rno	361677	5.04	30314.79	52.35	77.23	−0.281
*Strongylocentrotus purpuratus*	spu	756664	5.61	33741.14	60.18	68.27	−0.494
*Tribolium castaneum*	tca	664056	5.9	29687.86	42.98	85.75	−0.339
*Taeniopygia guttata*	tgu	100220909	5.03	30394.82	46.29	79.34	−0.268
*Takifugu rubripes*	tru	101078355	4.93	30363.69	45.7	80.8	−0.218
*Trichinella spiralis*	tsp	3457	4.78	27893.53	36.6	92.42	−0.247
*Xenopus laevis*	xla	443846	4.93	29983.24	47.91	77.21	−0.313
*Xenopus tropicalis*	xtr	448770	5.26	30069.42	43.11	76.47	−0.322

These data are representative from several taxa and would permit to infer about the evolutionary history of Tollip in diverse animal groups. Main protein features were inferred through diverse web servers from amino acid sequences.

All evolutionary analyses were carried out on the MEGA 5 software [24]. Maximum likelihood phylogenetic trees were obtained for Tollip using a Muscle alignment and G Blocks curation with default sets at PhyML 3.0 [25], using the most appropriate model of amino acid substitution and likelihood scores assessed by TOPALi V2.5 [26], [27]. The best model was determined by using the Akaike Information Criterion (AIC) [28], [29]. Supports for the nodes were evaluated by bootstrapping with 1000 pseudoreplicates.

The effect of reticulate evolutionary events was analyzed through a neighbor-net analysis [30] and converted into a splits graph using the drawing algorithms implemented in SplitsTree4 software – version 4.10 [31]. The neighbor-net method was based on the pairwise distance matrices of Tollip complete sequences alignment with deletion of gaps and non-informative parsimony sites; the matrices were calculated and corrected with the Poisson distribution model [32].

The isoletric point (pI) and molecular weight (MW) of each protein sequence was inferred with the Compute pI/Mw tool [33], the variability present in sequences was accessed through the Protein Variability Server [34], and the main protein characteristics as instability index (ININ), aliphatic index (AI) and GRAVY (grand average of hydropathicity) were evaluated with the ProtParam tool [35]. Tests of correlation between collected data and statistical treatments were made with GraphPad Prism version 5.0 software [36].

To check if selection affected the patterns of genetic diversity, we tested if the protein was under positive selection. Tajima’s D statistic [37] was calculated by testing the mutation neutrality hypothesis [38], as previously described by Coscollá and colleagues [39]. In order to investigate the presence of positively selected codons, the estimation of both positive and purifying selection at each amino acid sites was calculated from the ratio of non-synonymous to synonymous substitutions, ω, as previously described [40]. Analyses were conducted using the Selecton version 2.1 software [41], [42]. The significance of scores was obtained by using a Likelihood Ratio Test that compares two nested models: a null model that assumes no selection (M8a) [43] and an alternative model that does (M8) [44].

Several approaches were used to determine the extent of recombination in the Tollip data set. First, Tollip protein sequences previously aligned at Clustal W2 [45] were back-translated at BioEdit [46] package using standard genetic code, to normalize the codon frequencies and bring/make the comparisons more accurate. Once recombination eventually creates mosaic sequences in which evolutionary history at each site may be different. Then, GARD method [47] available in Datamonkey server [48] was also used to search for evidence of phylogenetic incongruences, and to identify the number and location of breakpoints corresponding to recombination events. In order to confirm GARD results, the recombination was assessed using a recombination cost ‘‘delta dirac’’ and mutation cost ‘‘Hamming’’, implemented in the Recco program [49]. The gap extension cost was fixed to 0.2 and the statistical significance of the analysis was obtained after 1000 permutations. Validation of the previously obtained results was performed with the six methods implemented in the RDP3 program [50]: RDP [51], GENECONV [52], BootScan [53], Maximum Chisquared Test [54], CHIMAERA [55] and Sister Scan [56]. The analysis was performed with default settings for the detection methods, a Bonferroni corrected P-value cut-off of 0.05, and a requirement that each potential event had to be detected simultaneously by four or more methods.

A tridimensional model was generated starting from hsa protein sequence, evaluating I-Tasser server [57], using default sets. This approach was used in order to assess the potential implications of our findings in the tertiary protein structure. Tests to search for ligands and hot spots in the protein were ran using the Profunc application [58] at PDBSum [59].

## Results and Discussion

**Figure 1:**
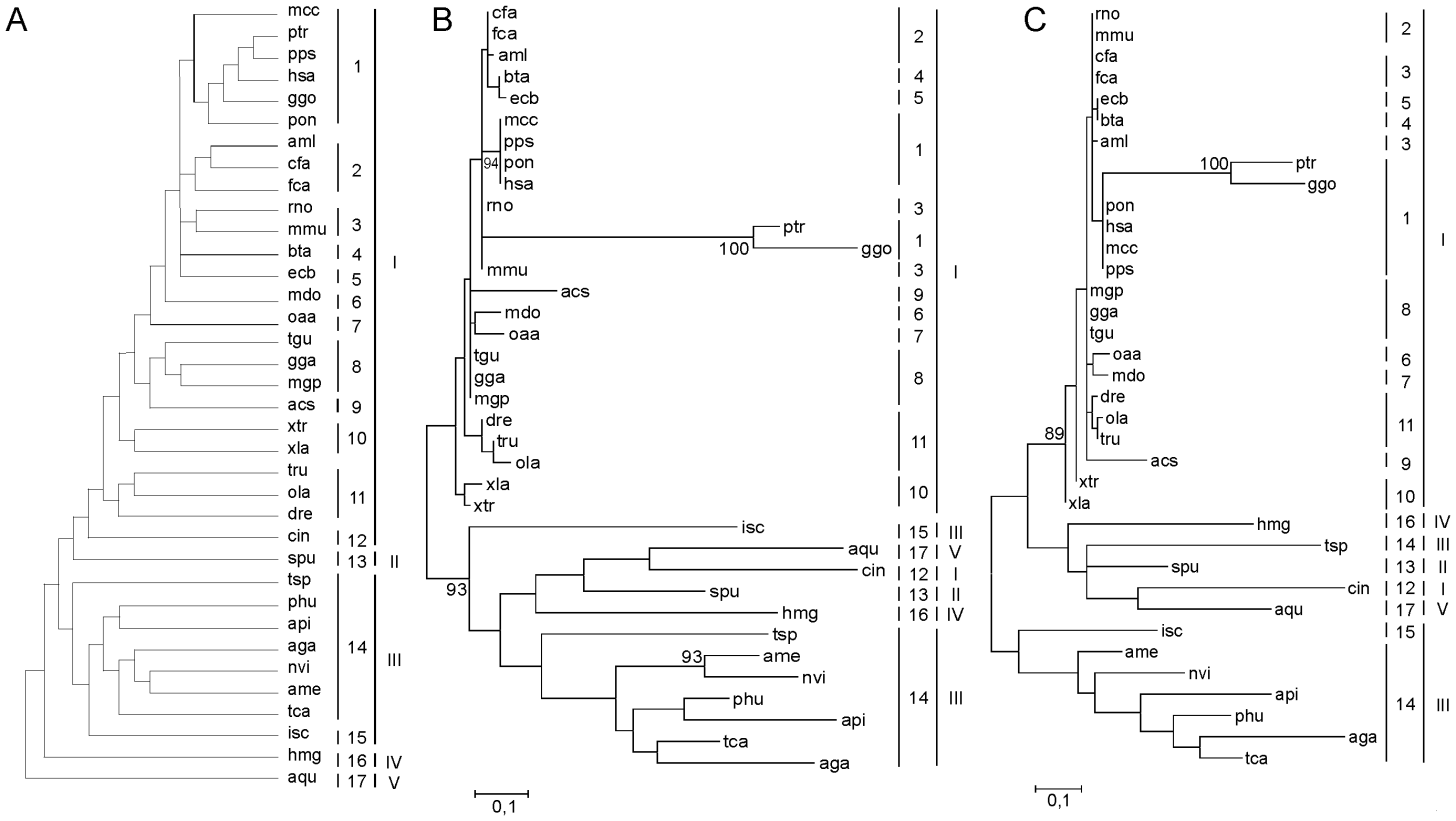
Molecular Phylogenetic analysis by Maximum Likelihood method. The evolutionary history of ToL (A), complete Tollip protein (B) and G-block cured Tollip protein (C) are shown. Trees B and C are drawn to scale, with branch lengths measured in the number of substitutions per site. The analysis involved 36 amino acid sequences. All positions containing gaps and missing data were eliminated. Group sorting was made in roman numerals (I - vertebrata, II - echinodermata, III - arthropod, IV - cnidaria, V - porifera) and the subgroups were coded in arabic numerals (1 - primates, 2 - carnivora, 3 - rodentia, 4 - bovidae, 5 - equidae 6 - marsupialia 7 - monotremata, 8 - birds, 9 - reptilia, 10 - amphibia, 11 - actinopterygii, 12 - ascidiacea, 13 - echinoidea, 14 - hexapoda, 15 - arachnida, 16 - hydrozoa, 17 - demospongiae).

The maximum likelihood composite trees generated are shown at Figure 1. The incongruent topology is evidenced by different branch sorting between phylogenetic trees of Tollip complete and G blocks trees, and between the current phylogenetic organization available at ToL. The most appropriate model for explaining the evolution of Tollip was found to be mtREV24 [60], [61] with following the parameters: BIC of 5424.48, AICc of 5006.19, lnL of -2432.61, Invariant sites n/a, and Gamma parameters of n/a.

The groups were split based on the Tollip protein sequence, confirming the evolutionary relatedness constructed in the ToL project. Despite this, a new phylogenetic array suggests other relationships between protein sequences of these animals. In Figure 1B, notice a node formed by subgroups 13 to 17, which include porifera (subgroup 17) and cnidaria (subgroup 16), together with bilateria, subgroups 13 to 15. The other branch is composed just by vertebrata, group I, which remains like a monophyletic group in all the trees, confirmed by bootstrap values in Figures 1B and 1C, respectively 81% and 89%. The separated groups, based in the Tollip sequence reinforces the evolutionary relatedness constructed in the ToL project. Although, other relationships between the vertebrates are suggested. Indeed, the group I suggests a characteristic function of the protein in order of higher levels of organisms complexity, requiring a less stable molecule for the modulation of information which will be discussed later. In the group I, the primates (subgroup 1) are in two arranges, in Figure 1B the ptr and ggo (bootstrap value of 100%) are far the other primates (bootstrap value of 94%) suggesting one difference in complete sequence, the non conserved sites difference ptr and ggo from the others. The conserved sites separated the two primates too, Figure 1C, the two sequences hold similarities between them in this molecular level.

In Figure 1C, there are two branches, one of them is formed by groups I to V and the other just by group III, except for subgroup 14. This configuration is due to the alignment of conserved sites in the sequences, showing the variability of Tollip sequences between different organisms. When the Tollip complete sequence was analyzed (Figure 1B), this configuration changed. The group III remains like a monophyletic group, the alignment of non conserved parts does not change significantly the branch, subgroup 14 returns to the group III and the subgroup 15 becomes paraphyletic relatively to group III. This allows us to consider group III as close enough to be relatively consistent throughout the entire analysis.

The average length of Tollip was 262 amino acids with a standard deviation of 64 amino acids; and the molecular weight average was 31.33 kDa with a standard deviation of 6.78 kDa. Through splits decomposition and analysis of alignment, after block curing, we could estimate the proportion of invariant sites as 68.54% and the segregant sites counted was 77 in amino acid base. These findings suggest a tendency of recurrent duplications and/or insertions, as well as deletions evidenced by variations in the length and mass of protein ranging between approximately 25% and 4.62 fold, respectively. But it is important to stress that the protein activity seems be intact or just slightly reduced, once its activity is essential to keep the health in animals. Tollip participates in several immune pathways, mediated or not by Myd88 [18], [19], modulating the responses and the loss or reduction of its affinity to molecular complexes made between it and several other compounds, like IRAK-1 [20]–[23], could trigger an exaggerated response of the immune system, leading to death in some cases. Though, there are studies, like Didierlaurent’s[62], which affirm that mice lacking Tollip become healthy and fertile.

Despite all identified polymorphisms and mutations of Tollip, we could not make any inferences about its role in the TLR-triggering activation of dendritic cells, without more in vitro and in vivo tests. Although, some studies [22], [62] have revealed that Tollip does not have a fundamental function in the TLR-triggering activation pathway of dendritic cells. Mutant mice lacking the tollip gene, when compared with wild-type mice, have been shown to not have significant differences. Therefore, mutations in key-residues for Tollip activity does not imply differences at the activation level of dendritic cells.

**Figure 2:**
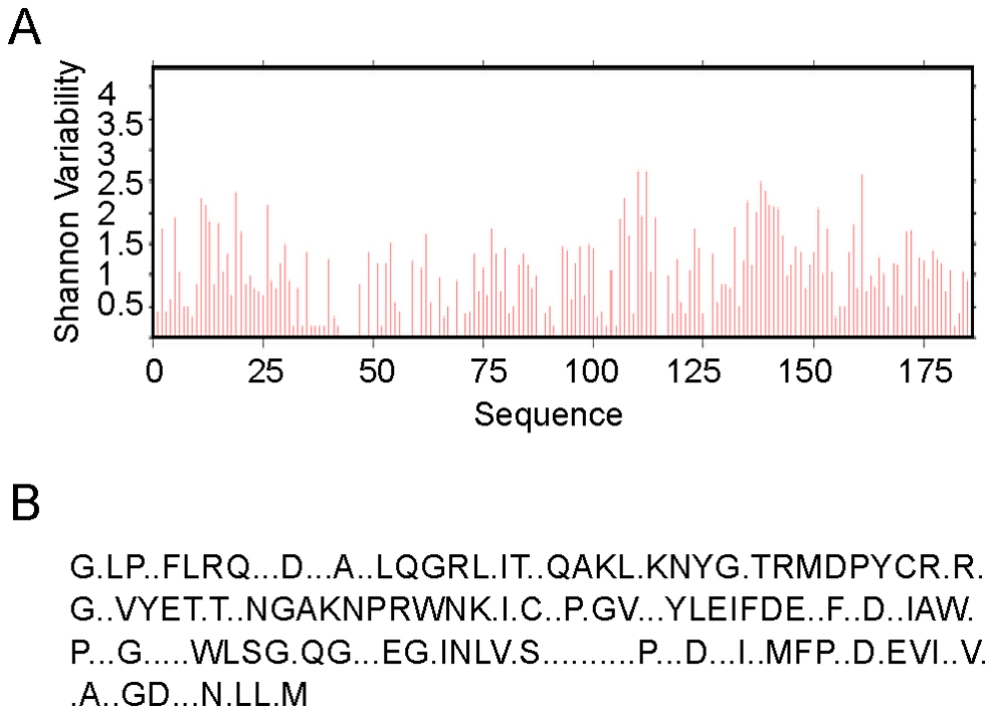
Variability of Tollip protein. The variability residue per residue measured with Shannon Index (A). Protein conserved residues are disposed at (B), with variable positions as ".", these positions reflect some essential molecular arrangement to Tollip function. All analyses were made with Tollip G-block cured, to avoid the gaps and non-informative parsimony site.

The characteristics of proteins were evaluated (Table 1) and the distribution of instability index (ININ), aliphatic index (AI) and GRAVY followed the normal distribution (P Kolmogorov Smirnov test >0.05). The molecular weight showed a statically significant correlation coefficient with aliphatic index (p = 0.014; r = −0.4065); another characters showed correlations between the aliphatic index and the instability index (p<0.001; r = −0.5829), and between GRAVY and the aliphatic index (p<<0.001; r = 0.6714). These data are shown in Table 2. The protein variability (Figure 2), was measured by the Shannon coefficient. We observed the Tollip G-block cured proteins, and the regions that have continuous conserved residues are probably responsible for the catalytic reactions or binding interactions.

**Table 2:** Correlation between main assessed protein characteristics of Tollip.

	MW	Instability Index	Aliphatic Index	GRAVY	pI	Arthropod	Primates
**MW**		**0.04**	**0.01**	0.21	0.36	0.19	0.07
**Instability Index**	**0.28**		**9.55E-05**	0.18	0.42	**0.01**	**0.001**
**Aliphatic Index**	**−0.41**	**−0.58**		**3.68E-06**	0.19	**0.01**	0.40
**GRAVY**	−0.14	−0.16	**0.67**		0.10	0.24	0.06
**pI**	−0.06	−0.04	−0.15	−0.22		0.08	0.16
**Arthropod**	−0.15	**−0.41**	**0.36**	−0.12	0.24		0.10
**Primates**	0.25	**0.51**	−0.04	0.27	0.17	−0.22	

These characters were evaluated under ANOVA 1 Factor test, followed by Bartlett's test for equal variances at 5% of significance. To evaluate the correlation coefficients and p-value was made using the Bonferroni's Multiple Comparison test at 5% of significance. R scores of correlation are shown under table's diagonal and p-value of each one are shown above table's diagonal. Significant values are in bold.

The variability of sequence lengths implies a complex organization of Tollip function or adjustment in diverse immune pathways. The standard deviation of the number of residues in a protein probably reflects a process of tertiary structure "stabilization", evidenced by increasing AI values, which were positively correlated (r = 0.366; p = 0.14) with arthropod group. Higher molecular weights showed higher hydrophobicity by AI (p = −0.407; r = 0.007) and GRAVY results showed similar findings, being correlated with AI too (r = 0.671; p = 3.7×10^−6^). These aliphatic residues seem to be essential to the evolving process of this protein. The ININ revealed by itself a tendency of accumulation of instabilities in all groups except the arthropods, once a positive correlation of these values was shown between primates group (r = 0.515, p = 0.001) and another negative was shown between arthropods and ININ (r = −0.413; p = 0.006). These tendencies are related with a hydrophobization of the entire molecule, which increase the molecule lifetime, being advantageous for their group due to rapid molecular ratio and molecular turnover [63]–[65].

Primates revealed just a tendency to increase instability of protein (ININ; r = 0.515, p = 0.001); this is related to lower half-life in this protein. It is in agreement with the higher available energy in these animals, in opposite that observed in arthropods or small animals. The cell environment of superior chordata can be very unstable which enables physiological reactions, with rapid and efficient beginnings and ends. The Tollip has sites for ubiquitination [22], which considerably reduces its life-time. In these groups of animals, it seems that Tollip has more sites available or sites with more affinity to ubiquitin.

**Figure 3:**
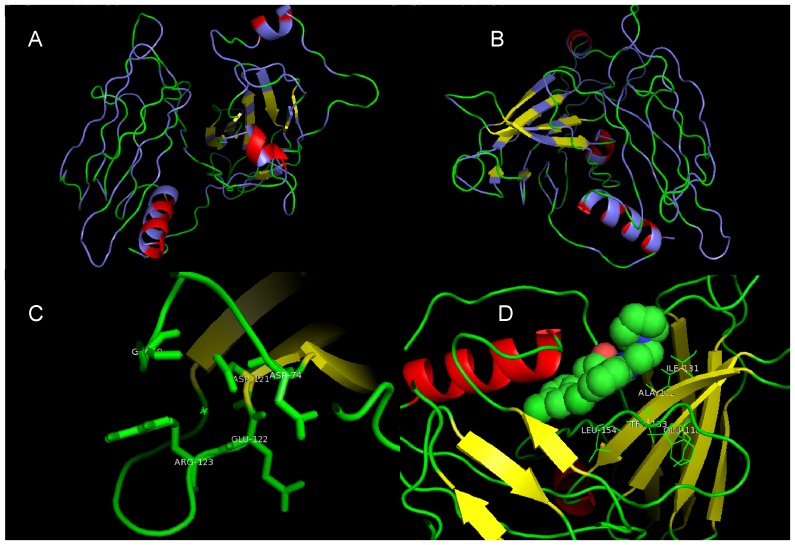
Human (hsa) Tollip tertiary structure. This structure was modeled at I-Tasser server, using default sets. It could be seen the aliphatic residues distribution along all sequence (A and B). The ligands are shown arranged at lateral chain of the right residues, Ca^2+^ (C) and 768 (D).

Starting from a virtual model of this protein, made from hsa sequence, at I-Tasser server using default variables had an estimated accuracy measured through TM-score of 0.34±0.12 and a RMSD of 14.1±3.8 Å. We tried to identify the pattern of hydrophobic pockets, but it was seen that aliphatic residual apolarity is homogenously distributed along the entire sequence (Figure 3.A and B). The main residues, evidenced by conservation (Figure 2.A), likely construct the reaction pockets and in the order of the modular characteristics of this protein [66], the alpha-helixes and beta-sheets are domains for binding to specific ligands. Some ions showed to be important to conservation of tertiary structure as calcium II (BS Score 1.34–1.39, RMSD 3.00, TM-Score 0.349). It contacts with G69-D74-D121-E122-R123 residues, as can be seen at Figure 3.C, that are relatively conserved. An organic compound, ligand 768 (1-(2,4-dichlorophenoxy)-3-{2-imino-3-[2-(1-piperidinyl)ethyl]-2,3-dihydro-1H-benzimidazol-1-yl}-2-propanol), was found to be a ligand which contacts with E118-I131-A132-W133-L154 residues, as can be seen at Figure 3.D, with a RMSD of 5.51, TM-Score of 0.25 and BS Score of 0.86. This ligand 768 is related with inhibition of calcium-dependent membrane binding activity of prothrombin and of factors Va, VIII and Xa of human coagulation pathway [67] by interaction with C2 domain. This interaction is consistent with Tollip modular criteria and its functions, revealing a potential need of Ca^2+^ to maintain the C2 domain structure and could be potentially inhibited by 768 ligand.

**Figure 4:**
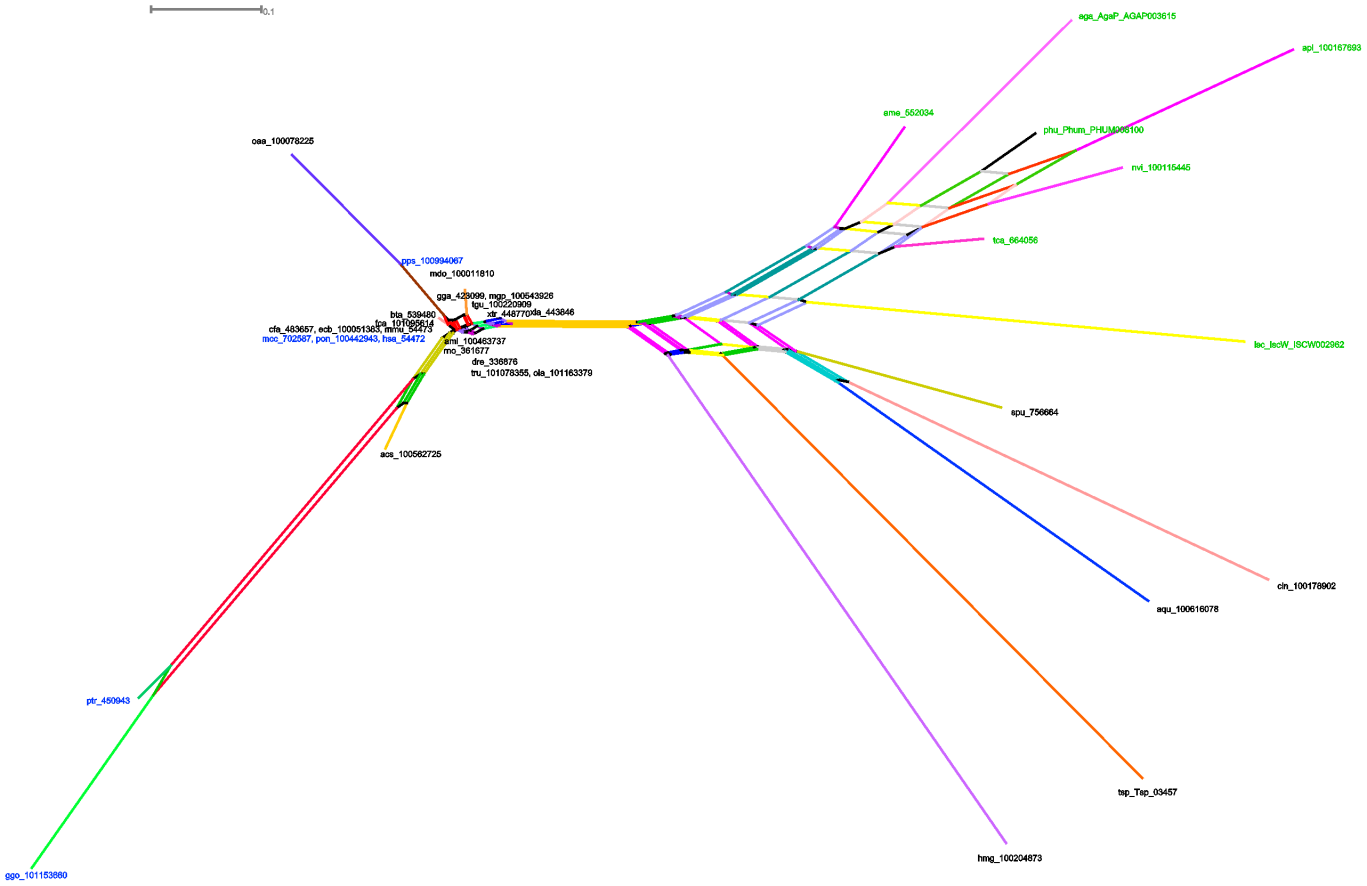
Splits graph of complete Tollip protein alignment. The parsimony uninformative sites, gaps and constant sites were excluded. There were 1000 pseudoreplicates performed as bootstraps to support the derivations, it was used as ProteinML distance correction the model mtREV24. Green operational taxonomic units represents arthropods groups, blue taxa represent primates and black represent the other groups.

Splits graph (Figure 4) using a neighbor-net analysis, excluding parsimony uninformative sites, gaps and constant sites, showed a concentrated reticulation in the evolutionary history of Tollip, mainly disposed in superior animals. These groups present a complex diversification history. Indeed, these incongruences evidenced by trees (Figure 1) reveal an interesting clustering pattern in this protein, stressing the diversification of arthropod in detriment of others. This division is due to the diverse pathogenic elements that eventually could enter in contact with the arthropod and the ubiquity of their presence in almost all possible environments (earth, air and water) could make this process more efficient and fast. The high bootstrap values evidence the strong support of presented nodes and clusters.

**Figure 5:**
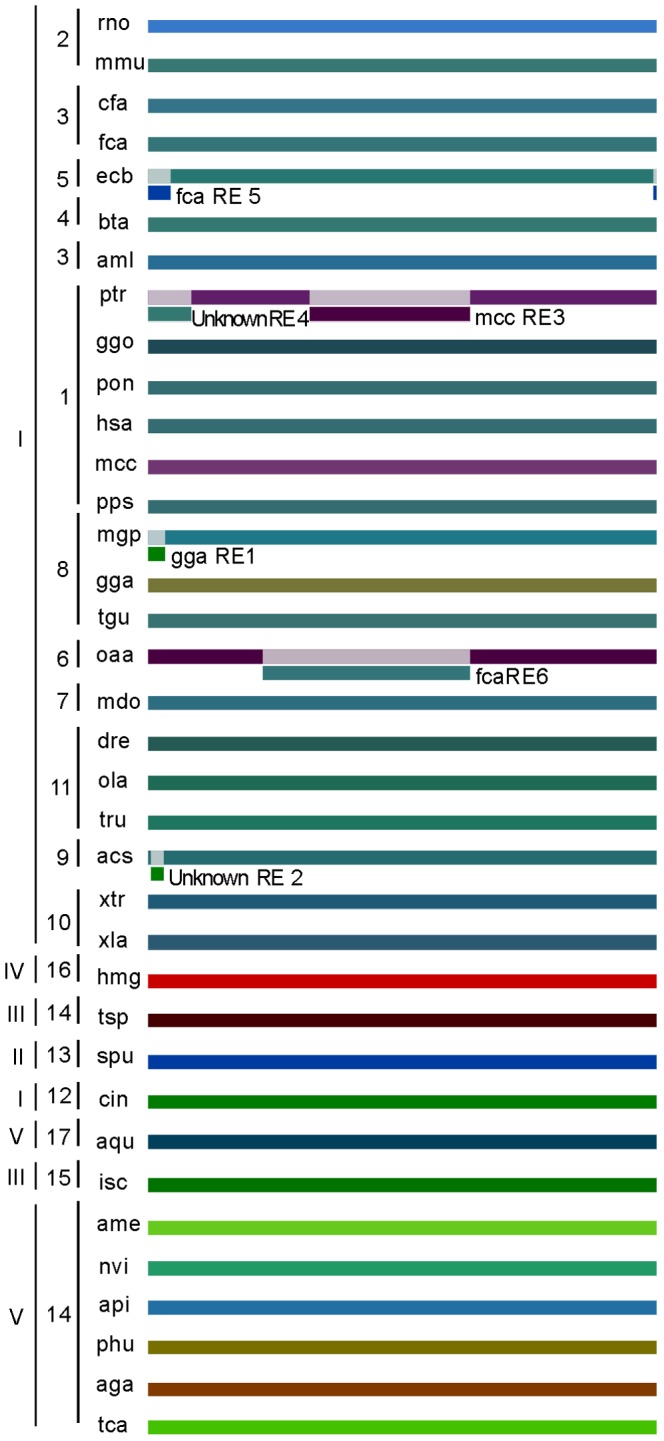
Recombinational events involved with Tollip evolution. Each sequence are represented by a color and the recombination is evidenced by donor. All analyses were evaluated with RDP and the most significant P value to support the findings are shown at Table 3.

GARD found at least 3 breaking-points with statistical significance (p<0.001, KH test) and these findings were supported by Recco analysis with 1000 bootstraps and by at least four different algorithms at RDP software (Table 3). RDP showed the same breaking-points (Figure 5) which comprised the hypothesis that recombinational events generated or could isolate some groups bringing new specific pathways. Some incongruences, as discussed before, could be explained by these events. Owing to a strange pattern of recombination found, the most probable hypothesis to support our findings is the horizontal transfer mediated by viruses or bacteria [68], that lived in the same environment of the two species, as some donors could not be identified with a high-threshold confidence level, these events could take part of very long and intrinsic evolutionary histories.

**Table 3:** Potential recombinant events identified in Tollip with RDP.

Recombinant Event (RE)	Recombinant	Major Parent	Minor Parent	Breaking Point	P - value	Recombination Detection Tests								
						RPD	GENCONV	BootScan	MaxChi	Chimaera	SiScan	PhylPro	LARD	3Seq
1	mgp	gga_423099 (100% similarity)	Unknown (cin_100178902)	839–31	4.544×10^−9^ (GENECONV)	1	2	-	2	1	-	-	-	-
2	acs	gga_423099 (92.9% similarity)	Unknown (cin_100178902)	5–30	2.990×10 ^−6^ (RDP)	1	2	-	2	1	-	-	-	-
3	ptr	mcc_702587 (99.6% similarity)	Unknown (bta_539480)	1–78	1.544×10 ^−7^ (SiScan)	-	-	1	1	-	1	-	-	1
4	ptr	aml_100463737 (90.2% similarity)	Unknown (oaa_100078225)	276–496	6.285×10^−28^ (RDP)	1	1	1	1	1	1	-	-	1
5	ecb	fca_101095614 (95.2% similarity)	Unknown (spu_756664)	817–45	1.627×10^−11^ (RDP)	1	1	-	1	1	-	-	-	1
6	oaa	fca_101095614 (97% similarity)	Unknown (api_100167693)	194–528	3.241×10^−21^ (GENECONV)	1	1	1	1	1	1	-	-	1

The molecular clock evaluated with the sequences (Table 4) showed that the sequences really presented different evolving patterns, where the sequences have increasing patterns of substitution when the complexity of the organisms become higher. These findings suggested that the Tollip evolutionary pattern is related with successive insertions and deletions that change the protein primary structure in order to bring less stable products; this is explained by the protein turnover that turns higher when the available energy and size of the animal increases [63]–[65].

**Table 4:** Results from a test of molecular clocks using the Maximum Likelihood method.

Complete Tollip Sequences				
	*lnL*	Parameters	(+G)	(+I)
With Clock	2648.884	35	n/a	n/a
Without Clock	2504.758	69	n/a	n/a

Curated G-Block Tollip Sequences				
	*lnL*	Parameters	(+G)	(+I)
With Clock	2375.702	35	n/a	n/a
Without Clock	2204.713	69	n/a	n/a

The molecular clock test was performed by comparing the ML value for the given topology with and without the molecular clock constraints under Poisson model [32]. The null hypothesis of equal evolutionary rate throughout the trees were rejected at a 5% significance level (*P*<<0.05). The analysis involved 36 amino acid sequences. All positions containing gaps and missing data were eliminated. There were a total of 88 positions in the final dataset. Evolutionary analyses were conducted in MEGA5 [24].

**Figure 6:**
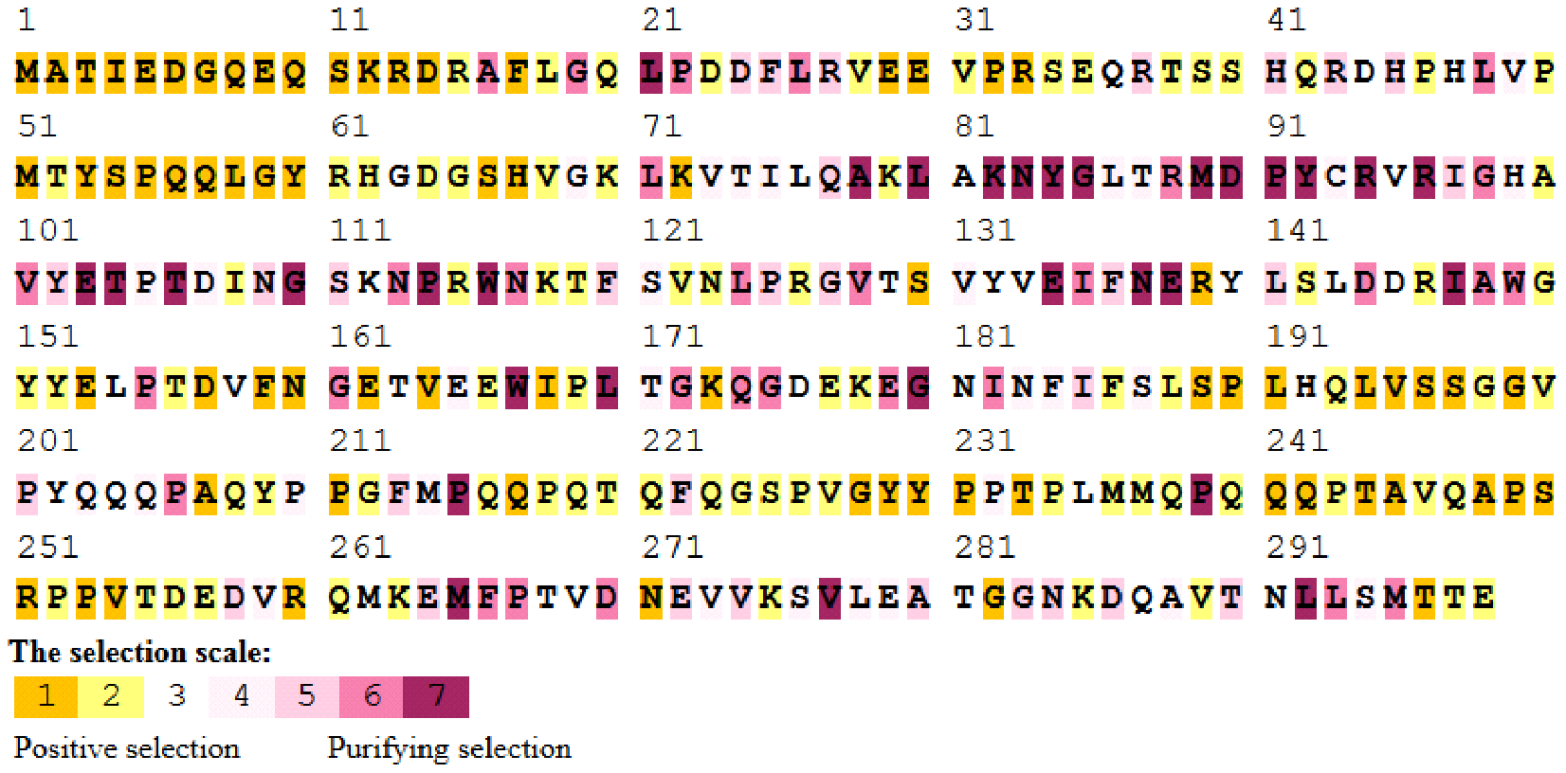
Positive selections operating in each codon of Tollip, evidenced by Selecton algorithm. There were used two models which were evaluated separately, M8 and M8a, where the last is referenced as null model.

Tajima's D statistic was 1.8226, meaning a tendency to positive selection. To assess the positive selection, we normalized the sequences using BioEdit through the back translation device, once the problem of codon preferences for each species could interfere in posterior analyses. To evaluate the results showed at Tajima's D statistic, the Selecton server was used and the results (Figure 6) showed a positive selection operating in almost all residues (49.33%) with statistical significance, M8 *versus* M8a as null model, evidenced by ΔLnL value of 179.6 (p<0.001).

These findings are consistent with the data presented by analysis of the segregant and conserved sites, where it was determined that the protein is variable and presents a very active process of restructuring. The protein domains from amino-terminal ends are under a high positive selection, indicating that these parts of protein are variable and become higher adaptative values with more variability. Several consecutive amino acids presented in the second domain in the sequence a relative conservation, including a tendency to negative selection. These residues are related with the activity of the protein. Indeed, they could participate in the Tollip protein-protein and protein-lipid interactions, crucial to the right performance on the pathway.

The modulatory activity of Tollip is directly related to their association with different intracellular factors, such as other proteins and calcium ions. We have noted that these residues responsible for such interactions suffer broadly neutral to negative selection, which in fact was obviously expected in order to keep their functionality.

Tollip polymorphisms were correlated with several human diseases like atopic dermatitis [69], inflammatory bowel disease [70], tuberculosis [71] and other. In atopic dermatitis (AD), Single-strand conformation polymorphism (SSCP) of the tollip sequence is correlated to AD. We could infer that amino acid exchanges of A (Ala) to S (Ser) occur at residue 222. Ser222 has a higher correlation to healthier controls (5.4%) when compared with Ala222 (2.7%) [69]. Residue 222 is occupied by A in 52.78% of sequences and is largely distributed in vertebrate sequences (subgroups 1–10), excluding oaa sequences. We found that it suffers a strong positive selection, through Selecton analysis (Fig. 6), shown as residue 245. In this case, seems that residue is conservated through vertebrata, subgroups 1 to 10, and we are inclined to believe that this is a trait which has became from an ancestor at amphibian level, and this mutation could be benefic to them too.

Tollip, in inflammatory bowel disease, suffers an inactivation that makes the intestinal epithelium unable to inhibit LPS-induced NF-kB activation [70], through a mutational amino acid exchange of lys150glu. In this sense, all primates present the residue K (Lys) at this position (except the ggo and ptr, which present R (Arg) and G (Gly), respectively), despite the common trend to present residue E (Glu) in other animals (55.56%). Residue D (Asp) in this position is important for insects (except for tsp and phu sequences, presenting E and Q (Gln) residues, respectively). This residue is under positive selection (position 166 at Selecton file, Figure 6). This mutation is apparently negative to health in primates and is a trait which was largely incorporated by other animals, reflecting a directional selection in this group, as occurs among insects.

In addition, a study involving tuberculosis (TB) and tollip [71] reveals that some synonymous polymorphisms or some that occur at noncoding regions (intronic regions or 3'UTR) could trigger different levels of risk of TB, not identifiable with protein analysis. This study also shows an association between minor homozygote of single-nucleotide polymorphism (SNP), named rs5743899, and a trend of reduced levels of Tollip mRNA in comparison with heterozygotes and major homozygotes, driving down the Tollip expression levels regulating cytokine response. Still, another SNP (rs3750920) was associated with increased levels of Tollip mRNA, providing protection to the organism against TB. The hypofunctional Tollip genotype has an association with increased levels of proinflammatory cytokines and increased risk of TB, as well as production of augmented proinflammatory cytokines. However, the assessed SNPs were related to synonymous variations or mutations in non-coding regions, and therefore, our data could not reveal any kind of correlation with that. Shah et al. [71] finds that tollip's effect in murine models are not applicable because tollip behaves differently in humans. There is a need for more research in this area.

## Conclusions

Tollip presents diverse evolutionary tendencies and several of them are indicating successive modifications in the protein structure, in order to stabilize the tertiary structure accumulating aliphatic residues. Primates generally have more unstable proteins, while arthropods have more stability at ININ, AI and GRAVY level. Size was not correlated with any groups and seems to be highly variable in all groups. In/del trends were saw as very frequent. The three dimensional structure analysis revealed the modular characteristic of this protein and the necessity of Ca^2+^ to keep the correct pocket of C2 domain. Ligand association studies revealed that 768 ligand probably could inhibit the Tollip activity. Positively selected residues were found in almost all domains, but a considerable part of them are relatively conserved, indicating a conservation of active pockets, which is consistent with maintaining protein right activity. The tested animal groups were differentially grouped, when studied with parsimonious and non-parsimonious residues, and revealed through molecular clock analysis that they present different selection and evolving speeds. The recombination supports diverse incongruences observed in the phylogenetic trees obtained with complete and cured Tollip data sets. There are no evidences that support a homogeneity in this immunologic pathway, once Tollip presented evolving trends that are not constant for all groups. Summarizing, some groups are highly evolutionary closed, as arthropods and primates, but when compared between them are totally non consistent.

In conclusion, differences in Tollip structure among vertebrates could be detected as well as changes occurring in the primary structure through evolutionary processes. Once these changes occur in Tollip's structure, the same must occur with other structures in the IL-1R and TLR pathway. Adaptive immunity is commonly seen as the most evolved aspect of the immune systems of these organisms, but our data suggest that innate immunity in vertebrates could also be evolving differently among the species in order to promote a better adaptation to their reality.
